# The impact of bacteria-derived ultrafine dust particles on pulmonary diseases

**DOI:** 10.1038/s12276-019-0367-3

**Published:** 2020-03-17

**Authors:** Jinho Yang, Eun Kyoung Kim, Hyeon Ju Park, Andrea McDowell, Yoon-Keun Kim

**Affiliations:** 1 Institute of MD Healthcare Inc., Seoul, Republic of Korea; 2 Department of Health and Safety Convergence Science, Graduate School of Korea University, Seoul, Republic of Korea

**Keywords:** Lung cancer, Immunopathogenesis

## Abstract

The relationship between ambient particulate matter exposure and health has been well established. Ultrafine particles (UFP) with a diameter of 100 nm or less are known to increase pulmonary disease risk. Biological factors in dust containing UFP can cause severe inflammatory reactions. Pulmonary diseases develop primarily as a result of chronic inflammation caused by immune dysfunction. Thus, this review focuses on the adverse pulmonary effects of biological UFP, principally lipopolysaccharide (LPS), and bacterial extracellular vesicles (EVs), in indoor dust and the pathophysiological mechanisms involved in the development of chronic pulmonary diseases. The impact of LPS-induced pulmonary inflammation is based primarily on the amount of inhaled LPS. When relatively low levels of LPS are inhaled, a cascade of immune responses leads to Th2 cell induction, and IL-5 and IL-13 released by Th2 cells contributes to asthma development. Conversely, exposure to high levels of LPS induces a Th17 cell response, leading to increased production of IL-17, which is associated with asthma, COPD, and lung cancer incidence. Responses to bacterial EV exposure can similarly be broadly divided based on whether one of two mechanisms, either intracellular or extracellular, is activated, which depends on the type of the parent cell. Extracellular bacteria-derived EVs can cause neutrophilic inflammation via Th17 cell induction, which is associated with asthma, emphysema, COPD, and lung cancer. On the other hand, intracellular bacteria-derived EVs lead to mononuclear inflammation via Th1 cell induction, which increases the risk of emphysema. In conclusion, future measures should focus on the overall reduction of LPS sources in addition to the improvement of the balance of inhaled bacterial EVs in the indoor environment to minimize pulmonary disease risk.

## Chronic lung disease: Hazards in indoor dust from bacteria

Tiny bacterial particles found in ultrafine airborne dust in homes and workplaces can trigger inflammation in the airways and increase the risk of developing chronic lung disease. Yoon-Keun Kim at MD Healthcare Inc in Seoul, South Korea, and co-workers review current understanding of the impact of ultrafine particles on disease development. Persistent, long-term exposure to molecules and extracellular vesicles from bacteria can trigger excess proinflammatory protein production in the body. Over time, this exacerbates immune dysfunction and increases the chances of developing conditions such as asthma, chronic obstructive pulmonary disease and lung cancer. Breathing in lipopolysaccharides shed from the membranes of certain bacteria may pose the greatest risk of developing chronic lung disease. The researchers call for urgent research to find ways of limiting lipopolysaccharides in indoor environments and optimizing air quality.

## Introduction

### Particulate matter

The air we breathe inside our homes, workplaces, schools, and public spaces is ubiquitously contaminated with a variety of types of particulate matter that comprise our indoor environment. Epidemiological studies worldwide have consistently demonstrated links between ambient particulate matter exposure and adverse health outcomes, including increased rates of respiratory and cardiovascular illness, hospitalization, and premature mortality^1–3^. Particles are usually defined based on their size: coarse particles are those with a diameter of 10 μm or less (PM_10_), fine particles are those with a diameter of 2.5 μm or less (PM_2.5_), and ultrafine particles (UFP) are those with a diameter of 100 nm or less, which are abundant in number but make only a minor contribution to the total mass^4^. UFP do not readily sediment or flocculate and thus have a longer retention time in the atmosphere than fine particles, allowing them to be carried long distances by the wind^5,6^. In terms of health hazards, fine particles are only absorbed by alveolar macrophages when they enter the lungs, whereas UFP are also absorbed by airway epithelial cells, which can trigger a potent airway inflammatory response. Moreover, unlike fine particles, UFPs are absorbed into the body and increase the risk of cardiovascular and neuropsychiatric disease. Due to this ability to more deeply infiltrate our normal lines of defense against foreign particulate matter, UFP are likely to be more harmful to health than fine particles^5–7^.

In addition, since most people spend ~87% of their time indoors^8^, interest in the correlation between indoor environments and health has been increasing^9,10^. Previous studies have suggested that indoor dust is a more important factor in health and disease than outdoor dust. Indoor dust has particularly significant effects on health, as it includes a wide variety of components, including PM_10_, PM_2.5_, nanoparticles, heavy metals, bacteria, fungi, and house dust mite-derived allergens. However, prominent organizations such as the World Health Organization (WHO) and Environmental Protection Agency (EPA) have not released specific regulations or guidelines in terms of the particular composition of pollutants or nanosized particles in the indoor environment. Rather, the existing regulations more broadly address the total bacteria count (TBC) and the acceptable levels of PM_10_, PM_2.5_, heavy metals, gaseous pollutants and other pollutants. Therefore, recent efforts have focused on exploring the specific causative factors and mechanisms involved in the effects of UFP as indoor air pollutants to understand the correlation between indoor air dust and disease.

### Impact of the biological factors in indoor dust on health

**Fig. 1:**
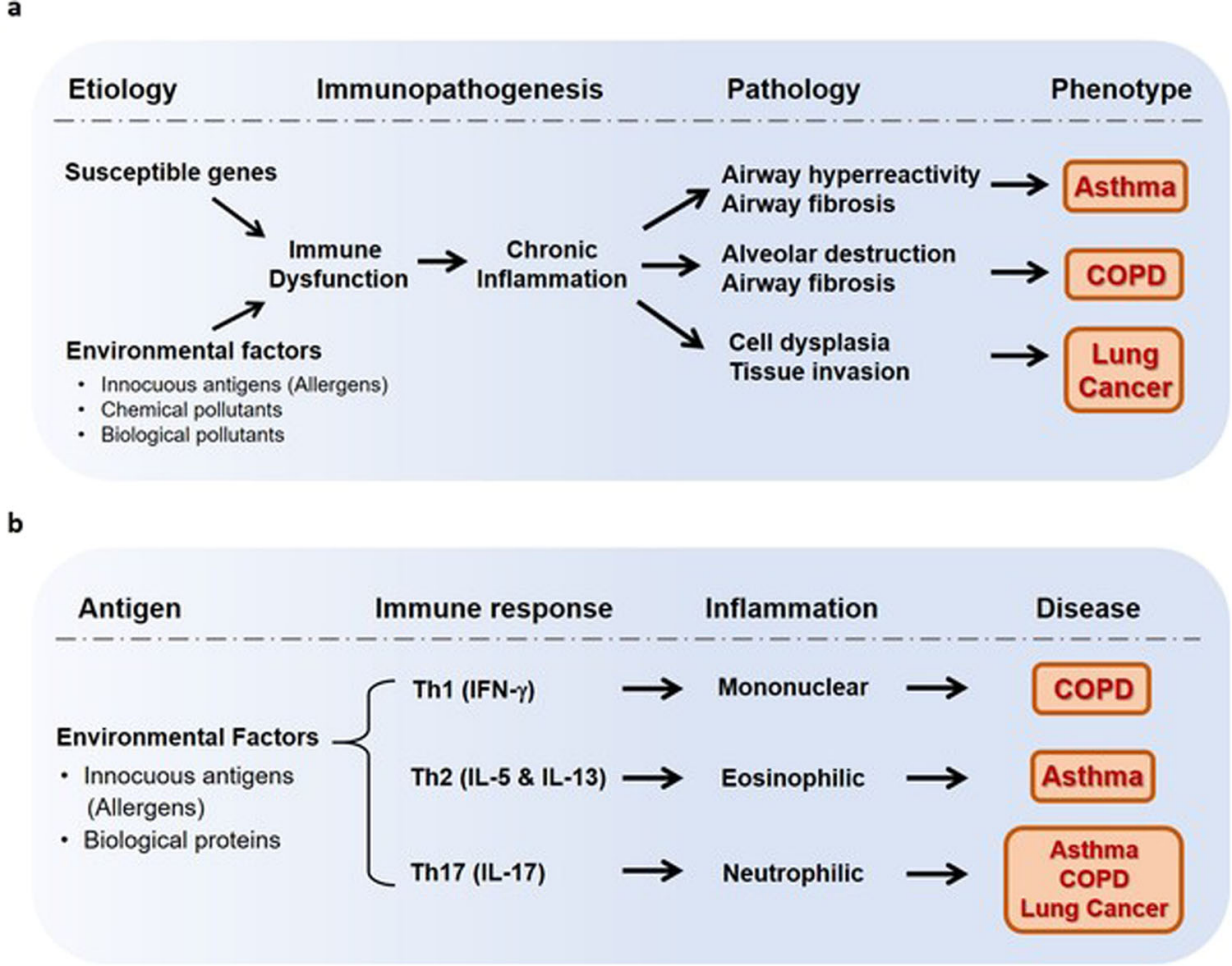
Asthma, COPD, and lung cancer development is associated with chronic inflammation, which is a result of immune dysfunction. Immune dysfunction is induced by susceptible genes and environmental factors, such as chemical and biological substances. Biological factors including allergens and biological proteins that act as antigens induce Th1, Th2, and Th17 cell immune responses. These immune responses lead to mononuclear, eosinophilic and neutrophilic inflammation and contribute to the development of COPD, asthma, and lung cancer.

Inhaled air pollutants are correlated with pulmonary disease and allergic diseases influenced by the environment, such as atopic dermatitis, asthma, and allergic rhinitis. Aside from air pollutants, smoking is also known to be an important factor in the development of chronic inflammatory pulmonary diseases such as asthma, chronic obstructive pulmonary disease (COPD), and lung cancer^11,12^. Furthermore, exposure to chemicals with harmful pharmacological effects is also a potential contributing factor to chronic inflammatory disease. However, the etiology of a large proportion of chronic inflammatory diseases in the lungs cannot be explained by the effects of such chemical factors alone. In addition, many COPD and lung cancer patients have no history of cigarette smoking^13^. Interestingly, the relationship between serum anti-dust extracellular vesicle (EV) IgG levels and the risk of asthma, COPD, and lung cancer is stronger than that for cigarette smoking^14^. Pulmonary diseases are a result of chronic inflammation induced by immune dysfunction stemming from the presence of genes conferring susceptibility and environmental factors, including chemical substances and biological factors. Asthma, COPD, and lung cancer generally develop as a result of pathology induced by chronic inflammation, e.g., airway hyperreactivity, airway fibrosis, alveolar destruction, cell dysplasia, and tissue invasion (Fig. 1a).

Biological factors such as allergens, viruses, and bacterial substances in dust can cause severe inflammatory reactions in certain cases. For example, bacterial factors might induce airway hypersensitivity due to exposure to protein antigens. Even when humans are exposed to very small amounts of bacterial factors, the body recognizes the antigens as harmful, and this can cause an inflammatory reaction. Clinical manifestations may vary depending on the type of inflammation that occurs in the airways^15–17^. Biological factors in indoor dust can induce immune dysfunction and chronic inflammation, leading to chronic inflammatory pulmonary diseases, including asthma and COPD^18,19^. Biological factors acting as antigens can differentially induce Th1, Th2, and Th17 cell immune responses, leading to the release of IFN-gamma, IL-5/IL-13, and IL-17; subsequently, these immune response factors lead to the activation of the mononuclear, eosinophilic, and neutrophilic pathways, respectively, and contribute to the development of COPD, asthma, and lung cancer (Fig. 1b).

Previous studies have demonstrated that Th17 is important for pathogen clearance and the support of host defense reactions and induces tissue inflammation in autoimmune diseases^20^. Functionally, angiogenesis and tumor growth are promoted by the overexpression of IL-17 in tumor cell lines in immune-compromised mice^21^. In addition, lung metastasis was shown to be suppressed by IL-17A deficiency or IL-17A blockade in tumor models^22^. Furthermore, IL-17 could directly promote the invasion of non-small cell lung cancer (NSCLC) cells, and an elevated IL-17 level in peripheral blood was shown to be related to the tumor-node-metastasis (TNM) stage^23^. These results indicate that tumor development is promoted by IL-17-mediated responses through the production of a microenvironment associated with tumor promotion and that a primary mechanism of tumor induction is the IL-17-mediated regulation of myeloid-derived suppressor cells (MDSCs)^24^. Regarding Th17 cells in lung cancer, recent studies have supported the role of Th17 cells in lung cancer pathogenesis. The levels of Th17 markers, such as RORC2, RORα4, IL-17A, IL-17A receptor, and VEGF, were significantly elevated in lung cancer patients^25,26^, and immune effector T cells (Teff) from small cell lung cancer (SCLC) patients included a large population of Th17 cells^27^. Furthermore, CD4 + -IL-17A + and CD8 + -IL-17A + cells were detected in lung adenocarcinoma tissues, whereas they were absent in normal tissues, suggesting the pathogenic role of IL-17A in lung adenocarcinoma^26^. Intranasal treatment with a neutralizing anti-IL-17A antibody in a lung adenocarcinoma model was also shown to reduce tumor growth^25^.

### Biological UFP in indoor dust

Biological fine particles contain microorganisms (bacteria and fungi) and contribute to ~5–34% of indoor air pollution^28^. However, a concrete scientific explanation of the underlying mechanisms and the extent to which indoor contaminants, including UFP, cause disease remains elusive. Microbes themselves cannot be classified as biological UFP, as the typical bacterium is over a micrometer in diameter, and pathogenic bacteria act primarily as infectious agents. However, nanometer-sized substances derived from microbes, such as lipopolysaccharide (LPS) and EVs, are common UFP in indoor dust. LPS has been characterized as having various sizes that are in the range of a few hundred nanometers; however, there have been numerous reports of nanosized (10–200 nm) LPS derived from bacteria^29–31^. Bacterial EVs are lipid bilayer vesicles that act as cell-to-cell communicators between bacterial cells or eukaryotic cells and are secreted during cell proliferation and death^32,33^. The endotoxin LPS is a cell wall component of gram-negative bacteria that is ubiquitous in our living environment^34^. LPS, which directly affects the development of disease, is both embedded in the cell membrane of gram-negative EVs and detached from the cell membrane in the soluble fraction (SF). The amount of LPS in dust SF was greater than that in dust EV; however, the levels of TNF- α and IL-6 induced by dust EV were significantly higher than those induced by dust SF^35^. Thus, this review focuses on the adverse pulmonary effects of biological UFP in indoor dust, such as LPS and bacterial EVs, and the pathophysiological mechanisms involved in the development of chronic pulmonary diseases such as asthma, COPD, and lung cancer.

## Lipopolysaccharide as a biological UFP

### Association between LPS and pulmonary diseases based on epidemiological studies

The endotoxin LPS is a cell wall component of gram-negative bacteria that is ubiquitous in our living environment. LPS is a contaminant in various organic dusts and environments that support gram-negative bacterial growth. Exposure to LPS induces the production of pro-inflammatory and immune-modulating factors via TLR4^34,36^. Furthermore, LPS is known to be a biological factor commonly derived from bacteria that is associated with chronic inflammatory pulmonary disease^13^. A significant and growing body of literature has reported on the association between endotoxin exposure and pulmonary disease, including asthma, COPD, and emphysema^37^.

Airborne endotoxin exposure has been linked with occupational respiratory diseases in work environments associated with high concentrations of organic dust, such as agricultural sites, cotton mills, breweries, and waste processing sites^38^. Interestingly, it has been reported that the LPS concentration in dust rather than the dust itself is positively correlated with decreased pulmonary function^39^. Epidemiological studies suggest that endotoxin exposure is positively associated with an increased risk of asthma and asthma severity in both adults and children^40^. Furthermore, atopic asthma has also been positively associated with early childhood exposure to endotoxins through the transmission of infectious disease^41^. Similarly, a higher abundance of airway microbiota and increased LPS levels have been identified in patients with neutrophilic asthma^42^. Altogether, these epidemiological findings suggest that exposure to LPS via inhalation may be positively correlated with respiratory disease.

### Pathophysiological mechanisms of LPS involved in the development of pulmonary disease

Although efforts to determine the concrete etiological basis of pulmonary disease are ongoing, several studies have confirmed that LPS is a causative factor in respiratory disease and other chronic inflammatory illnesses. In a mouse model, an allergen challenge was performed in three ways: ovalbumin (OVA) with no LPS, OVA with 0.1 µg of LPS, or OVA with 100 µg of LPS. While treatment with OVA, a protein commonly used to stimulate allergic reactions, did not induce inflammatory responses, OVA combined with LPS was reported to induce inflammatory responses, suggesting that LPS is an essential element of the inflammatory response associated with immune dysfunction in the airways. Moreover, OVA combined with a low dose of LPS led to Th2 cell responses, and OVA combined with a high dose of LPS was shown to induce Th1 inflammatory responses. These findings confirmed that the inflammatory immune response in the airway is differentially induced depending on the amount of LPS present in the indoor air environment^43^. Our group’s research findings also demonstrated that different forms of asthma can be induced in an in vivo mouse model depending on the amount of LPS. Animal models indicated that allergen sensitization with a low dose of LPS (0.1 µg) induced type 2 asthma phenotypes, such as airway hyperresponsiveness, eosinophilic inflammation, and allergen-specific IgE upregulation. Allergen sensitization with 10 µg of LPS-induced asthma phenotypes such as airway hyperresponsiveness and Th17 cell-mediated neutrophilic inflammation^18^.

**Fig. 2:**
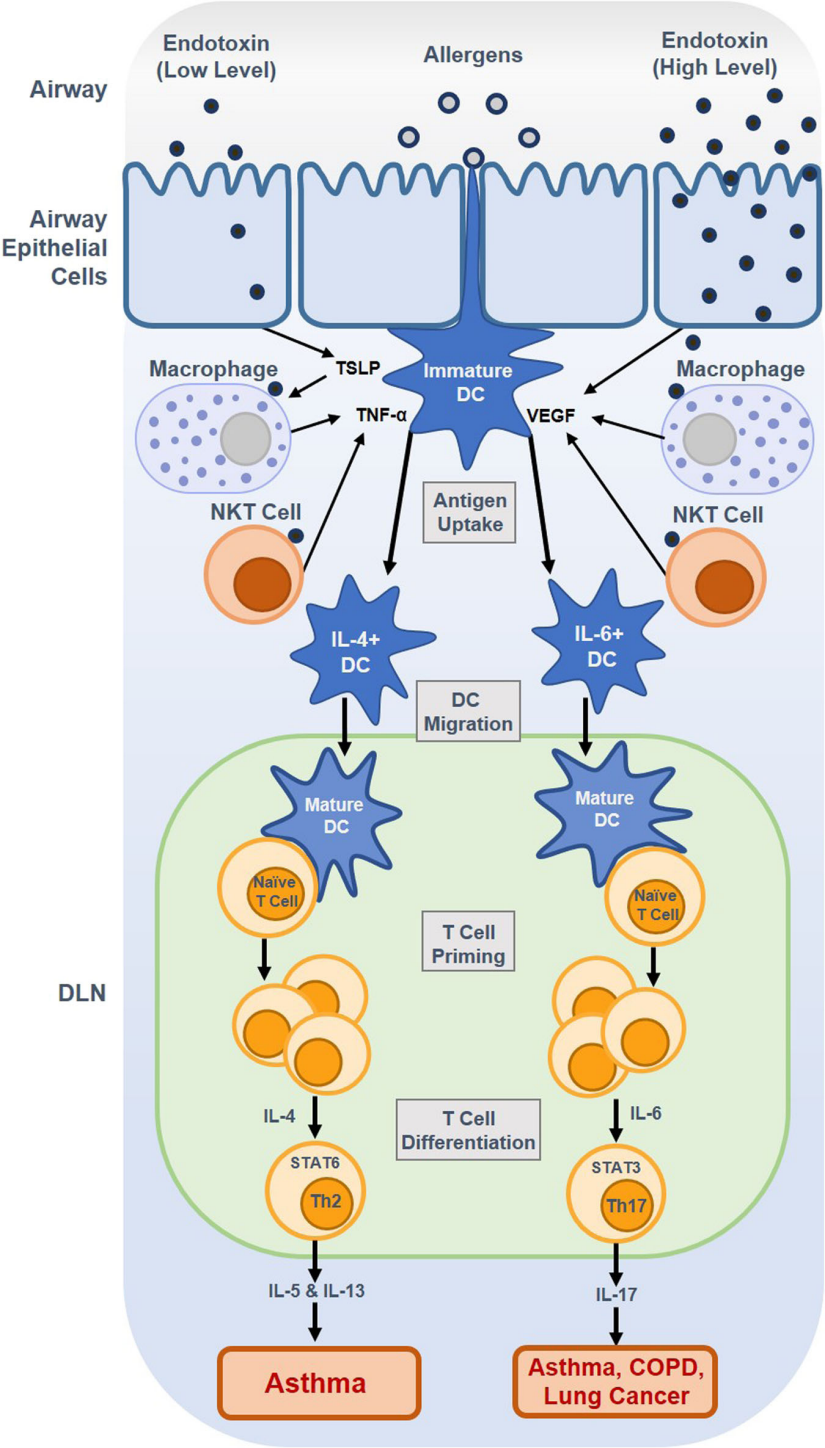
Low level-lipopolysaccharide (LPS) in the airway is absorbed into airway epithelial cells and immune cells, such as macrophages, natural killer T (NKT) cells, and dendritic cells (DCs). Exposure to low levels of LPS in the airway is associated with Th2 immune response and Th2 cytokines, such as IL-5 and IL-13, which contribute to the development of asthma. High levels of LPS in the airway induce vascular endothelial growth factor (VEGF). High levels of LPS is related with Th17 immune response and increased Th17 cytokines, such as IL-17, that contributes to the development of asthma, COPD, and lung cancer.

Specific immunological responses have been shown to vary in response to differing levels of LPS in the airway, which can be generally classified according to two immunological mechanisms: Th2 and Th17 immune responses (Fig. 2). It has been well established that low doses of inhaled LPS induce a Th2 cell response. Furthermore, when a combination of a low dose of LPS and allergens enters the airway through inhalation, only LPS is absorbed into airway epithelial cells and immune cells, such as macrophages, natural killer T (NKT) cells, and dendritic cells (DCs). Airway epithelial cells activated by a low dose of LPS induce the secretion of pro-inflammatory mediators, such as thymic stromal lymphopoietin (TSLP). TSLPs derived from epithelial cells both function in DC activation and enable DCs to polarize naïve T cells, leading to the production of proallergic Th2 cytokines such as IL-5 and IL-13^44^. Furthermore, this immunological pathway leads to the production of (TNF-α) in surrounding cells in response to macrophages and NKT cells activated by TSLP or low levels of LPS. TNF-α released by the surrounding immature dendritic cells then induces the increased expression of costimulatory molecules and IL-4 in dendritic cells^18^. These mature dendritic cells then migrate to the draining lymph nodes (DLN) and induce T cell proliferation and differentiation into Th2 cells through the production of IL-4, activating the signal transducer and activator of transcription 6 (STAT6) signaling pathway^45^. Through the STAT6 pathway, chronic inflammatory signaling is initiated as Th2 cells are induced to release IL-5 and IL-13, which are pro-inflammatory cytokines associated with the development of eosinophilic asthma and eosinophilic bronchitis.

Conversely, exposure to high levels of inhaled LPS is primarily associated with the Th17 cell response. High-dose LPS exposure induces vascular endothelial growth factor (VEGF) release from surrounding cells, including airway epithelial cells, macrophages, and NKT cells^46^. VEGF is produced during the LPS-induced innate response and acts as a potent mediator of vascular remodeling and angiogenesis in the lungs^47^. VEGF induces DC maturation and expression of costimulatory molecules via the vascular endothelial growth factor receptor 1 (VEGFR1)-dependent pathway. Through this pathway, IL-6-expressing DCs are then upregulated, and mature DCs are transferred to the DLN. Upon T cell proliferation, the excess T cells differentiate into Th17 cells via the IL-6 and STAT3 signaling pathways. Through the immunological response to high levels of airborne LPS, Th17 cells are induced to secrete IL-17, contributing to the development of serious pulmonary diseases such as neutrophilic asthma, COPD, and lung cancer^18,46,47^.

## Bacterial EVs are a key etiological agent of chronic pulmonary inflammatory diseases

Bacterial EVs are vesicles encased in a lipid bilayer containing transmembrane proteins, cytosolic proteins, LPS, and nucleic acids that range from 20 to 100 nm in diameter. EVs are ubiquitous in all bacteria and are commonly referred to as outer membrane vesicles (OMVs) in Gram-negative bacteria and membrane vesicles (MV) in Gram-positive bacteria^32,33^. After oral administration of bacteria-derived EVs, the EVs are absorbed through the intestinal barriers and transported to various organ systems, including the liver, adipose tissue and skeletal muscle^48^. As the EV membrane is embedded with surface ligands that interact with receptors on target cells, EVs can attach to and modify the physiological state of recipient cells^49,50^. Furthermore, bacterial EVs have recently been shown to be involved in the development of a wide variety of diseases, including cancer, diabetes, atopic dermatitis, and meningitis.

### Association between EVs and pulmonary diseases based on epidemiological studies

A variety of epidemiological evidence has suggested a strong link between the presence of bacterial EVs in dust in the indoor environment and chronic pulmonary illness. For example, a previous clinical study reported that while only ~5% of patients with allergic rhinitis or atopic dermatitis and healthy subjects showed sensitization to bacterial EVs present in apartment dust, over 50% of patients with childhood asthma were shown to be sensitized to EVs in indoor dust. This finding indicates the particularly significant role that EVs in indoor dust play in the development of childhood asthma-associated immune responses^35^. Higher serum anti-dust EV IgG antibody levels were also reported in patients with noneosinophilic asthma, COPD, and lung cancer than in healthy controls^14^. Interestingly, adjusted multiple logistic regression showed that the significant increase in anti-dust EV IgG antibody levels in patients with pulmonary disease compared with those in healthy controls was an independent risk factor for asthma, COPD and lung cancer irrespective of more typically associated factors, such as smoking history, age, and sex^14^. Altogether, while research on the impact of bacterial EVs in indoor dust on pulmonary disease is in its early stages, preliminary findings indicate that bacterial EVs pose a serious risk in terms of asthma, COPD, and lung cancer incidence.

### Pathophysiological mechanism of microbial EVs involved in the development of pulmonary disease

Our group was the first to report the presence of large amounts of bacterial EVs in indoor dust collected from beds in apartments^32,51,52^. Significantly, these indoor dust bacterial EVs were found to be mainly derived from pathogenic bacteria. The microbial EV composition of indoor dust is dominated by bacteria from the genera *Pseudomonas*, *Acinetobacter*, *Enterobacter*, and *Staphylococcus* (Manuscript in submission). We also demonstrated that prolonged exposure to bacterial EVs in inhaled indoor dust induces significant airway inflammation that leads to severe asthma-like responses as well as emphysema. The induction of emphysema is of particular concern, as it is known to be a major factor in the development of irreversible airway obstruction, including COPD^35^.

**Fig. 3:**
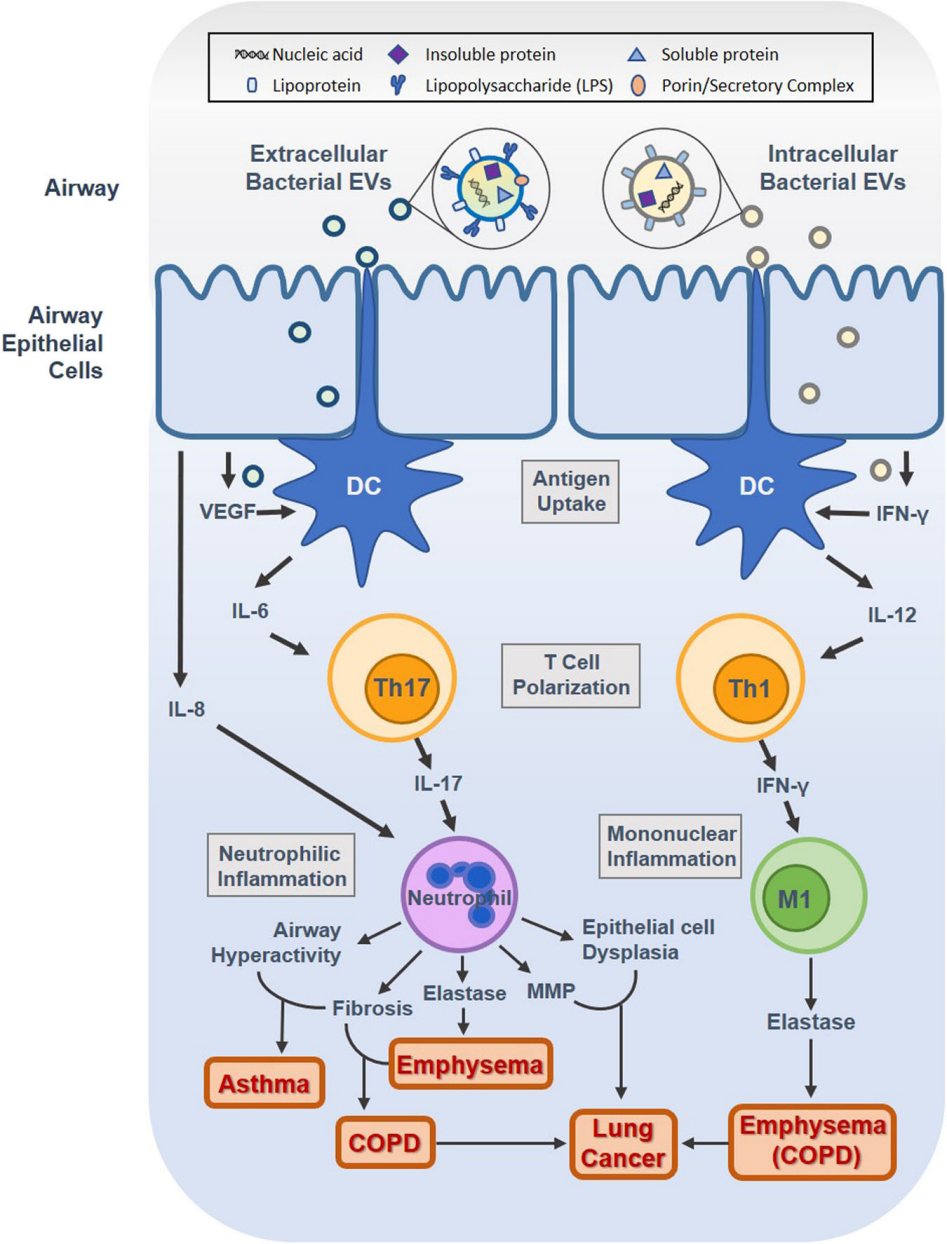
Bacteria-derived extracellular vesicles (EVs), especially Gram-negative outer membrane vesicles (OMVs), induce Th17 immune response and lead to neutrophilic inflammation. Intracellular bacteria-derived EVs, such as Gram-positive mycobacterial membrane vesicles (MVs) induce Th1 response and lead to mononuclear inflammation. These immune responses contribute to the development of asthma, emphysema, COPD, and lung cancer.

Airway exposure to microbial EVs triggers two main pathophysiological mechanisms based on whether the parent cell is extracellular vs. intracellular: the Th17 response is induced in response to extracellular bacteria-derived EVs, especially Gram-negative OMVs, and the Th1 response is induced in response to intracellular bacteria-derived EVs, such as Gram-positive mycobacterial MVs (Fig. 3). Specifically, extracellular bacterial EVs generally induce neutrophilic inflammation via IL-17 release by polarized Th17 cells, while intracellular bacterial EVs cause mononuclear inflammation through IFN-γ produced by polarized Th1 cells. The neutrophilic inflammatory response induced by pathogenic bacterial EVs generally leads to airway hyperreactivity and fibrosis that contributes to asthma development, while the combination of increased elastase production and fibrosis induced by neutrophilic inflammation can lead to COPD. Furthermore, epithelial cell dysplasia and induction of matrix metalloproteinase (MMP) expression associated with neutrophilic inflammation can lead to lung cancer, and an increase in COPD incidence increases lung cancer risk. Meanwhile, intracellular bacterial EVs, such as mycobacterial EVs, lead to Th1 polarization and subsequent IFN-γ-induced mononuclear inflammation, leading to increased elastase production in alveoli and thus causing emphysema.

Our previous studies have shown that repeated airway exposure over four weeks to Gram-negative *Escherichia coli* (*E. coli*)-derived EVs in mice induced IL-17A-dependent neutrophilic inflammation and emphysema in conjunction with elastase upregulation^53^. Intraperitoneal injection of EVs derived from intestinal *E. coli* induced host responses that resemble clinically relevant conditions, such as systemic inflammatory response syndrome (SIRS), that were characterized by piloerection, eye exudates, hypothermia, tachypnea, leukopenia, disseminated intravascular coagulation, dysfunction of the lungs, hypotension, systemic increases in TNF-a and IL-6 production, and lethality^54^. *Pseudomonas aeruginosa (P. aeruginosa)* often contributes to pulmonary diseases such as cystic fibrosis, and its EVs increase pulmonary inflammation through activation of the TLR2 and TLR4 pathways. *P. aeruginosa*-derived EVs induce pulmonary inflammation via dose-dependent increases in the chemokines CXCL1 and CCL2 and the cytokines IL-1β, TNF-α, IL-6, and IFN-γ and increases in neutrophils and macrophages in vivo^55^. Moreover, indoor dust, which contains many components of bacteria, has been associated with both Th1 and Th17 responses, which induce neutrophilic pulmonary inflammation^35,56^. As bacteria-derived EVs can affect distal host cell sites^14^, these vesicles might have significant effects throughout the body. These results suggest that the pathogenicity of bacterial EVs is dependent on the substances contained in the EVs.

Although Gram-negative OMVs are better understood and characterized than Gram-positive MVs, several studies have uncovered immunological responses to some common Gram-positive MVs in the airway. For example, mononuclear cell-induced inflammatory responses associated with Gram-positive EVs produced by pathogens such as *Staphylococcus aureus (S. aureus)*, a typical gram-positive bacterium, have been implicated in atopic dermatitis-like skin inflammation^51,52^. *S. aureus* EVs increase IL-6, IL-17, serum IgG1, IgE, TSLP, MIP-1α, and eotaxin levels in addition to increasing the recruitment of mast cells and eosinophils in the skin^51^. Furthermore, repeated exposure to *S. aureus* EVs in the airways induced both Th1 (IFN-γ) and Th17 (IL-17) immune responses in addition to increasing neutrophilic pulmonary inflammation primarily through TLR2 engagement^57^. While the immune response to airway microbial EVs can be broadly categorized based on the intracellular vs. extracellular type of the original cells, the specific immune responses to different types of bacterial EVs are summarized in Table 1. While various immune responses have been reported for different species of microbial EVs, common airway immune responses can be identified based on the Gram-type of indoor dust microbial EVs. For example, common airway Gram-negative EVs, such as those derived from *P. aeruginosa, E. coli*, and *Acinetobacter baumanii (A. baumanii)*, all increased IL-6 levels and neutrophilic activity. Meanwhile, Gram-positive EVs, such as those derived from *S. aureus* and *Faecalibacterium prausnitzii (F. prausnitzii)*, have been reported to commonly increase IFN- γ levels.

**Table 1:** Immune responses to bacterial extracellular vesicles.

Bacterial EV	Immune response	Reference
**Gram Negative OMV**		
*Pseudomonas aeruginosa*	Dose-dependent gene and protein expression of MIP-2, TNF-a, IL-1B, and IL-6 in vitro.	Ellis, et al.^58^
	Induction of pulmonary inflammation via dose-dependent increases in CXCL1, CCL2, IL-1β, TNF-α, IL-6, and IFN-γ as well as increases in neutrophils and macrophages in vivo.	Park, et al.^55^
*Escherichia coli*	Induction of systemic inflammatory responses via increases in serum TNF-α, IL-6, and IFN-γ and increases in lung permeability and dysfunction in vivo.	Park, et al.^54^
	Induction of emphysema through IL-17a-mediated neutrophilic inflammation in vivo.	Kim, et al.^53^
	Induction of systemic and lung inflammation via increased TNF-α and IL-6 levels in serum and bronchoalveolar lavage fluid in vivo.	Jang, et al.^59^
*Acinetobacter baumannii*	Intratracheal administration of EVs elicits expression of IL-1β, IL-6, IL-8, MIP-1α, and MCP-1 in lungs in addition to vacuolization, detachment of epithelial cells and neutrophilic infiltration.	Jun, et al.^60^
	Hemorrhage, necrosis, and infiltration of polymorphonuclear leukocytes in lung tissue 48 h after intratracheal infection of EVs in vivo.	Jin, et al.^61^
**Gram Positive MV**		
*Staphylococcus aureus*	Increases in IL-6, IL-17, serum IgG1, IgE, TSLP, MIP-1α, and eotaxin levels in addition to the recruitment of mast cells and eosinophils in a dose-dependent manner.	Hong, et al.^51^
	Induction of neutrophilic pulmonary inflammation through Th1 and Th17 cell responses in vivo.	Kim, et al.^57^
*Faecalibacterium prausnitzii*	Upregulation of the anti-inflammatory cytokines IL-10, TFG-β2, and IL-1RA and downregulation of the pro-inflammatory cytokines IL-6, TNF-α and TNF-β in a lung cancer cell line.	Jafari, et al.^62^

*OMV* outer membrane vesicles, *MIP* macrophage inflammatory proteins, *TNF* tumor necrosis factor, *IL* interleukin, *CXCL* chemokine (C-X-C motif) ligand, *CCL* Chemokine (C-C motif) ligand, *MV* membrane vesicles, *Ig* immunoglobin, *s-Ig* secretory Ig, *IFN* Interferon, *TSLP* thymic stromal lymphopoietin, *TFG* transforming growth factor

## Conclusion

Prolonged exposure to biological UFP contained in indoor dust has been recently demonstrated to induce a variety of innate and chronic immune responses that can lead to the development of a variety of respiratory diseases. Generally, bacterial UFP can be segregated into two classes: very fine LPS particles and bacterial EVs. Previously, LPS has been targeted as the key biological UFP contributing to indoor dust, and recent studies have highlighted the influence of bacterial EVs. For example, it has been shown that treatment with Gram-negative EVs elicits stronger pro-inflammatory immune responses than that with isolated LPS, primarily due to the necessity of vesicle proteins for macrophage internalization^58^. Such findings suggest that while Gram-negative bacterial OMVs contain LPS, other components of bacterial EVs influence the activation of pro-inflammatory innate immune responses by the innate immune system. Furthermore, EVs from Gram-positive bacteria lacking LPS are similarly able to differentially illicit both powerful pro- and anti-inflammatory immune responses, emphasizing the complex effects bacterial EVs have on chronic pulmonary disease risk.

As understanding of the significant health risks posed by inhaled ultrafine biological particles begins to coalesce within the scientific community, it is necessary to implement appropriate measures to improve the air quality of indoor environments to reduce chronic pulmonary disease risk. Although LPS as an isolated ligand plays a more straightforward dose-dependent etiological role in pulmonary disease, the complex, dynamic nature of bacterial EVs requires a more holistic approach towards the optimization of the indoor environment. While further metagenomic and proteomic assessment of bacterial EVs in a wider variety of indoor environments and vulnerable populations is required, preliminary findings indicate that efforts to improve the indoor dust bacterial EV balance should focus on the normalization of environments with heavy amounts of Gram-negative contamination. In conclusion, future measures should focus on the overall reduction of LPS sources in addition to the improvement of the overall balance of inhaled pathogenic and beneficial bacterial EVs in the indoor environment to minimize pulmonary disease risk.
